# Functional MRI Investigation of Ultrasound Stimulation at ST 36

**DOI:** 10.1155/2020/6794013

**Published:** 2020-02-22

**Authors:** Yarui Wei, Ling Mei, Xiaojing Long, Xiaoxiao Wang, Yanjun Diao, Benedictor A. Nguchu, Sheng Hu, Yanming Wang, Haibo Yu, Bensheng Qiu

**Affiliations:** ^1^Centers for Biomedical Engineering, University of Science and Technology of China, Hefei 230027, Anhui, China; ^2^Shenzhen Key Laboratory for MRI, Shenzhen Institute of Advanced Technology, Chinese Academy of Sciences, Shenzhen 518055, China; ^3^Shenzhen International TCM Training Center, Shenzhen Hospital of Traditional Chinese Medicine, Shenzhen 518002, China

## Abstract

**Background:**

Clinical and experimental data suggest that ultrasound stimulation (US) at acupoints can produce similar effective treatment compared to manual acupuncture (MA). Although the brain activation to MA at acupoints is investigated by numerous studies, the brain activation to US at acupoints remains unclear.

**Methods:**

In the present work, we employed task state functional magnetic resonance imaging (fMRI) to explore the human brain's activation to US and MA at ST 36 (*Zusanli*) which is one of the most commonly used acupoints in acupuncture-related studies. 16 healthy subjects underwent US and MA procedures in an interval of more than one week. On-off block design stimulation was used for the recording of fMRI-related brain patterns.

**Results:**

Both US and MA at ST 36 produced activations in somatosensory and limbic/paralimbic regions (postcentral gyrus, insula, middle prefrontal cortex, and anterior cingulate cortex). Only US at ST 36 produced a significant signal increase in the inferior parietal lobule and decrease in the posterior cingulate cortex, whereas MA at ST 36 produced a significant signal increase in the lentiform nucleus and cerebellum.

**Conclusions:**

Our results indicate that US may be a possible noninvasive alternative method to MA due to its similar activation patterns.

## 1. Introduction

Acupuncture has been shown to be effective in relieving Bell's palsy, pain, and anxiety and modulating the physical function of the human body [1–4]. Acupuncture involves stimulation with needles inserted in the skin at definite depth, eliciting a composite of sensations, such as soreness, numbness, fullness, dull pain, and warmness. These sensations, collectively called *de qi*, are considered to be related to clinical efficacy in traditional Chinese medicine. However, inserting the needle to a specific meridian point at definite depth has the risk of infection and requires more training and experience. For these reasons, some alternative modalities of manual acupuncture (MA) have been developed to stimulate the acupoint, such as ultrasound, laser, and electricity [5–7]. Ultrasound stimulation (US) is a noninvasive method that applies proper ultrasound energy to the acupoint through an ultrasound transducer. US has the advantages of preventing infection, dispelling the patient's fear to acupuncture, and setting stimulation frequency and intensity objectively and quantifiably. Several studies indicate that US at ST 36 (*Zusanli*) and other acupoints can produce similar effective treatment compared to MA [8, 9].

Although the brain activations to MA and its other modalities at acupoints are investigated by numerous studies [6, 10–17], the brain activation to US at acupoints using functional magnetic resonance imaging (fMRI) is still not substantially explored. Previous fMRI researches demonstrate that needling at acupoints activates both cortical and subcortical brain structures, particularly in primary and secondary somatosensory cortices (SI and SII) and limbic/paralimbic regions [13, 17]. Napadow et al. examine the brain response to MA and low- and high-frequency electroacupuncture (EA), demonstrating that low-frequency EA produces more widespread fMRI signal alterations than high-frequency EA [18]. Apart from EA, laser acupuncture (LA) has also been evaluated to modify the cerebral activation [16]. Bai [19] compares the therapeutic effects for the pain relief among different alternative modalities, and the results indicate that all of these modalities are effective to release pain. Moreover, US obtains the best therapeutic result than others, which is expected due to the fact that ultrasound has better capability of penetrating energy into the body deeply and noninvasively and spatial specificity than electrical and laser impulses. Previous studies only use the peripheral blood flow volume [20], blood pressure, and pulse rate [21] to explore the biological effect of US at acupoints. There is no research to investigate the brain activation to US at acupoints and compare whether the brain activation of US at acupoints is similar to that of MA at acupoints.

Many studies, respectively, report similar therapeutic effects of US and MA at acupoints. US at ST 36 obviously reduces the systolic pressure, diastolic pressure, and pulse of hypertension patients [21]. MA at ST 36 and other acupoints also reduces the blood pressure in patients using antihypertensive drugs [8]. In addition to that, US at CV 4 (*Guanyuan*) increases the estrogen secretion function and promotes the development of follicles in menopausal rats [22]. MA at SP 6 (*Sanyinjiao*) and CV 4 has been discussed for the treatment of diseases caused by estrogen deficiency [23]. We hypothesized that the coherent hemodynamic signal of these modalities may be observed.

Therefore, the current study aimed to conduct a preliminary investigation of brain activation in response to US and MA at ST 36 of the healthy individuals' left leg. ST 36 is one of most important and commonly used acupoints for treating various medical conditions in scientific research and clinical trials.

## 2. Materials and Methods

### 2.1. Subjects

This study was approved by the Institutional Review Board of Shenzhen Institute of Advanced Technology (SIAT-IRB-120215-H0125) and registered in the Chinese Clinical Trial Register (ChiCTR1900026207), and subjects' written informed consent was obtained before the experiment. 16 right-handed acupuncture-naive healthy volunteers (8 females and 8 males) aged 21–30 years (mean 24.1 years) participated in the experiment. The subjects' medical histories got reviewed to eliminate participants with any intimation of endocrinal, neurological, and psychiatric illnesses.

### 2.2. Experimental Paradigm

All subjects were recruited to participate in two fMRI scanning sessions, and in each session, the subjects received only one type of stimulation: US or MA. The two sessions were randomized and separated by a minimum of one week. All stimulation operations were conducted at ST 36 on the left legs by a specialized and licensed acupuncturist. In MA, the acupuncturist used a MRI-compatible disposable sterile titanium alloy needle of 0.32 mm in diameter and 50 mm in length (Suzhou Acupuncture & Moxibustion Appliance Co. Ltd., China). The needle was inserted 20–30 mm in depth, depending on the size of the tibialis anterior muscle of the subject. The acupuncturist manipulated the needle within the tolerance of the subject until the subjects obtained the *de qi* sensation without sharp pain. After achieving *de qi* sensation, the needle was rotated bidirectionally twice per second in the stimulation period. For US, the stimulator (Shengxiang High Technology Co. Ltd., Shenzhen, China) at a frequency of 824 kHz and a power of 1.75 W, which is of endurable intensity, was used to obtain the *de qi* sensation without sharp pain. The used frequency and power are close to the standardized mode and concord to those used in the previous study [5]. Aligned to the location of the acupoint and fixed on the skin using the tape, the ultrasonic transducer was connected to the power generator placed in the scanning room with an MRI-compatible cord.

**Figure 1:**
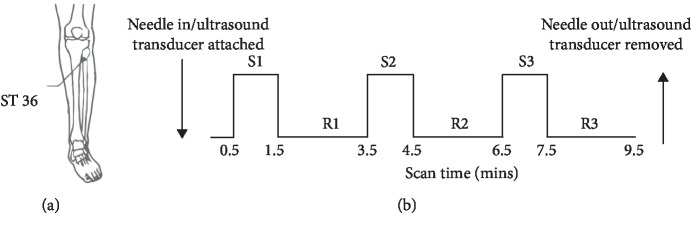
Experimental paradigm. (a) Location of ST 36. (b) Flowchart of the experimental procedure. S: stimulation; R: rest; mins: minutes.

The total scan time for each session was 9.5 minutes (Figure 1). At the beginning, the acupuncture needle was inserted in the skin or the ultrasound transducer was attached to the skin at ST 36. After the *de qi* sensation without sharp pain was achieved, scanning commenced with a rest period of 0.5 minutes. This was followed by 3 cycles of needle stimulation or US, and each cycle consisted of a 1-minute stimulus and 2-minute rest period. Finally, the acupuncture needle or the ultrasonic transducer was removed from the acupoint.

Prior to the experiment, volunteers had full understanding of acupuncture sensations that might arise in the US or MA course. Volunteers obliged to categorize their sensations into soreness, dull pain, numbness, fullness, and warmness and adopted a 10-point scale to self-rate the intensity of the sensations they had felt (0 = no sensation, 1–3 = mild, 4–6 = moderate, 7–8 = strong, 9 = severe, and 10 = unbearable sensation) after US or MA. Paired Student's *t*-test was performed to compare the strengths of sensation between two modalities.

### 2.3. fMRI Scanning

3T Scanner (MAGNETOM Trio Tim, Siemens, Erlangen, Germany) equipped with a 12-channel phase array head coil performed MR imaging. Ears plugged with sponge and eye goggles reduced noise and light interference, respectively, to subjects at the imaging session. Using the 3D Turbo FLASH sequence, we obtained high-resolution T1-weighted images with the following parametric settings: TR/TE = 1.9 s/2.53 ms, FOV = 250 mm, slice thickness = 1 mm, flip angle = 9°, and matrix size = 256 × 256. Imaging of BOLD fMRI was possible using the T2^*∗*^-weighted gradient-echo pulse sequence with the following parametric settings: TR/TE = 3 s/30 ms, FOV = 200 mm, slice thickness = 3 mm, flip angle = 90°, gap = 20%, matrix size = 64 × 64, and 39 sagittal slices.

### 2.4. Image Processing and Statistical Analysis

Firstly, data were preprocessed by using SPM12 (Statistical Parametric Mapping; http://www.fil.ion.ucl.ac.uk/spm/). The preprocessing encompassed the following operations: slice timing correction, realignment, coregistration to T1-weighted images, normalization to the MNI standard space, and spatial smoothing by a 6 mm FWHM Gaussian kernel. In our work, data with a translational motion >1.5 mm or a rotational migration >0.5° got dismissed from further steps. Eventually, 13/16 datasets qualified for proceeding analysis.

Secondly, one-sample *t*-test and *P* value maps corresponding to responses of the brain due to US or MA were computed by using the 3dttest++ module of AFNI software. The task of regulating and control of the false positive discovery rate was assigned to multiple-comparison correction which is integrated in achieved Monte Carlo simulation. We only focused on the results of each modality.

## 3. Results

### 3.1. Psychophysical Responses

**Figure 2:**
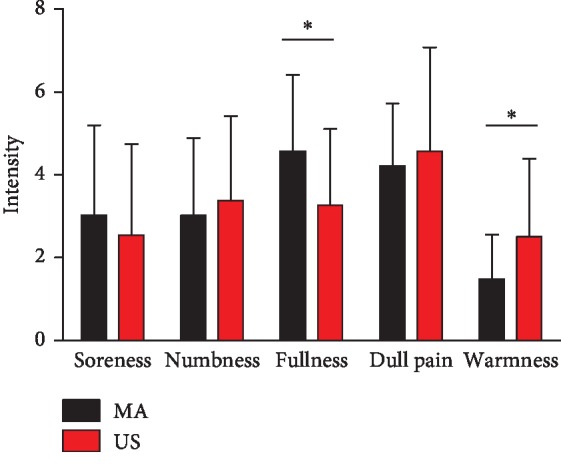
Psychophysical sensation rating during manual acupuncture (MA) and ultrasound stimulation (US) at ST 36. The error bars represent the standard error (*n*=13). ^*∗*^*P* < 0.05.

Most of the *de qi* sensation intensities were similar between two modalities (Figure 2). No significant difference was found between the two modalities with respect to the sensation of soreness, numbness, or dull pain (*P* > 0.05). MA induced significantly stronger sensing of fullness than US (*P* < 0.05), whereas US induced significantly stronger sensing of warmness than MA (*P* < 0.05). No participant reported a sharp pain in US or MA.

### 3.2. Brain Activation to US

**Figure 3:**
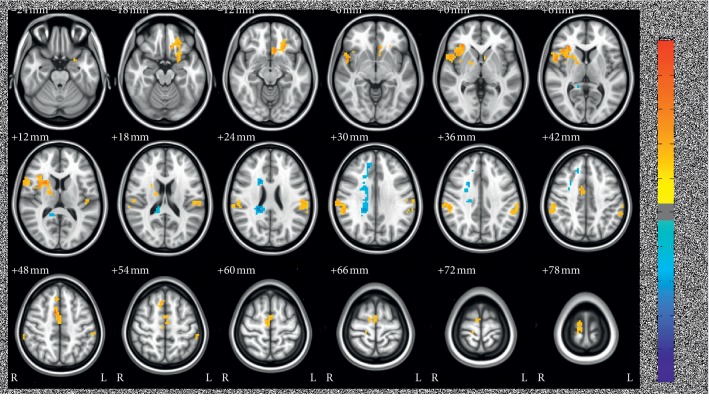
Group analysis results of activation in response to ultrasound stimulation at ST 36.

Table 1 and Figure 3 display outcomes of group analysis to US. The regions under this description become highly activated subsequent to US: right insula, right anterior cingulate cortex, left postcentral gyrus, left middle frontal gyrus, and right inferior parietal lobule. The right posterior cingulate gyrus remains deactivated in spite of US stimulation. The aforementioned results conformed to the multiple-comparison correction per Monte Carlo simulation with *P* < 0.05, alpha <0.05, and cluster size >156.

### 3.3. Brain Activation to MA

**Figure 4:**
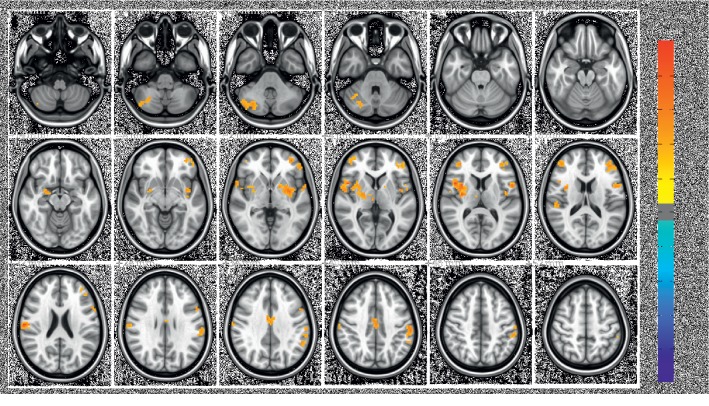
Group analysis results of activation in response to manual acupuncture at ST 36.

Table 2 summarizes group analysis results of MA, whereas Figure 4 illustrates these results pictorially. The following regions demonstrated high activations: right insula, bilateral middle frontal gyrus, bilateral postcentral gyrus, left lentiform nucleus, right cerebellum, left precentral gyrus, and right anterior cingulate gyrus. The presented results were corrected by using Monte Carlo simulation, with *P* < 0.02, alpha <0.05, and cluster size >61.

Interestingly, it can be observed that both US and MA at ST 36 assent to similar activated regions with substantial signal intensity: insula, middle prefrontal cortex, anterior cingulate cortex, and postcentral gyrus.

## 4. Discussion

In this study, we have shown that there is similar brain activation under the stimulation of these two modalities. Both US and MA at ST 36 produce activations in the somatosensory area and limbic/paralimbic regions, including the insula, the middle frontal gyrus, the cingulate cortex, and the postcentral gyrus. To our knowledge, we are the first to examine the brain activation of US at ST 36 using fMRI, which supplies a neurophysiological evidence for clinical efficacy of US at the acupoint.

By applying stimulation at ST 36, both modalities produced significant activations in the somatosensory area (postcentral gyrus) and limbic/paralimbic regions (insula, middle frontal gyrus, and cingulate cortex). These regions comply with those in previous researches. Activations in somatosensory areas have been frequently reported in previous acupuncture researches [13, 14], usually regardless of the acupoint chosen, primarily due to the fact that the postcentral gyrus, known as SI, together with the inferior parietal lobule, involves in the integration of sensory information such as the sense of touch. Apart from somatosensory areas, activations in limbic/paralimbic regions including the insula, the cingulate cortex, and the middle frontal gyrus were also observed in both modalities. The insula directly involves in the interoceptive awareness of the body states including heartbeat [24] and emotion [25]. The systolic pressure, diastolic pressure, and pulse of hypertension patients have been obviously reduced after US at ST 36 [21]. Activation in the middle frontal gyrus may have been reflecting activation related to a particular part of the mid-dorsolateral prefrontal cortex [26] which is well known in monitoring of the information of working memory and might reflect the mental tracking (monitoring) during stimulation of the leg. In addition to the prefrontal lobe, the anterior cingulate cortex is known to deal with error detection and confliction monitoring, as well as for its role in pain modulation [27, 28]. These areas might form the intrinsic neural circuit and play a central role in the antipain, antianxiety, and other therapeutic effects under US or MA [29]. Although more pathological studies of comparing brain response to US with that to MA are still needed to give grounds for this inference, our study still provides experimental evidence for the existence of similar activated regions and patterns in two modalities.

US may act as a noninvasive alternative to MA due to its similar hemodynamic activation patterns, which may also exist at other acupoints. Our results demonstrated that there existed similar brain activation between US and MA at ST 36. As described in Introduction, many studies indicate the similar effective treatment after US and MA at ST 36 and other acupoints [5, 8, 9, 21–23, 30, 31]. Both US and MA can improve the estrogen secretion function to treat the disturbance in estrogen levels [22, 23]. Blood flow volumes of both US and MA groups increase gradually and show a significant increase at 180 seconds after stimulation at LR 3 (*Taichong*) [5]. From the perspective of the physical mechanism of ultrasound acting on the tissue, the biological effect of US mainly includes thermal, mechanical, and thixotropic effects, which are similar to the composite sensations of MA [9]. Moreover, the previous study indicates that the US at LI 4 (*Hegu*) can elicit *de qi* sensation intensities similar to the MA [32], which is consistent with our results. Both US and MA are likely to induce the degranulation of mast cells [31, 33], which exist broadly at the acupoint [34] and play a role of analgesia after MA [31]. The blood pressure of hypertension patients is obviously reduced after US or MA at ST 36 [8, 21]. ST 36 is one of the most important and commonly used acupoints for treating various medical conditions in traditional Chinese acupuncture [35]. Therefore, the similar hemodynamic activation patterns of two modalities may exist at other acupoints.

US at acupoints may be a possible treatment method for motion- and emotion-related diseases due to brain activations in the somatosensory area and limbic/paralimbic regions. MA, a somatosensory conditioning stimulus, induces beneficial motion-related cortical plasticity in patients with carpal tunnel syndrome [36]. The beneficial motion-related cortical plasticity might also exist after the treatment of US at acupoints due to similar brain activation in the somatosensory area. Although there are satisfying effects due to US at the carpal tunnel in patients with mild to moderate carpal tunnel syndrome [37], the better treatment effect might be specially got after the treatment of US at related acupoints. In addition, the insula acting as a limbic-related cortex showed widespread activation to US and MA at ST 36. The previous study implicates that the insula is associated with self-referential processing in lower emotional awareness in patients with functional movement disorders [25]. Moreover, acupuncture at acupoints is a promising treatment for emotion [38]. However, there is a need for exploring the association between emotion-related diseases and US at acupoints.

It is also worth noting that the two modalities still show slight differences in the sensation intensity and activation pattern of some brain region. Slight differences in the sensation intensity between two modalities are comparable to the finding from the previous study, which indicates that US might reduce levels of non-*de qi*-related sensations, such as sharp pain and anxiety [32]. It can explain that the MA produces the activations of the lentiform nucleus and cerebellum, whereas US does not. Although the subjects do not report sharp pain, the activations of the lentiform nucleus and cerebellum in MA may relate to the anxiety sensation [39, 40]. Deactivation of the posterior cingulate gyrus in US at ST 36 may be explained by the fact that the sensations of fear and anxiety decrease in the US [41].

However, there are still several limitations in this study: First, we did not measure the brain activation of the sensory control at ST 36 in healthy subjects. Acupuncture can modulate the activity within specific brain regions [42]. Therefore, we only focused on whether the brain activation of two modalities is similar or not, and the resting periods in our study design may be seen as baseline. Moreover, the present preliminary results require validation with a larger sample size and further investigation.

## 5. Conclusions

US and MA at ST 36 produce similar brain activations in the somatosensory area and limbic/paralimbic regions, including the insula, middle frontal gyrus, cingulate cortex, and postcentral gyrus, on healthy individuals. Our results indicate that US may be a possible noninvasive alternative method to MA due to its similar hemodynamic-signal patterns.

## Figures and Tables

**Table 1:** Group analysis results of activation in response to ultrasound stimulation at ST 36.

Regions	Voxels	Side	Peak MNI coordinate (mm)			*T* value (peak intensity)
			*X*	*Y*	*Z*	
Insula	395	R	24	11	8	5.81
Posterior cingulate cortex	244	R	21	−34	23	−5.06
Anterior cingulate cortex	238	R	9	5	47	6.28
Postcentral gyrus	187	L	−57	−28	20	4.47
Middle frontal gyrus	161	L	−22	34	−19	3.00
Inferior parietal lobule	158	R	54	−34	32	3.65

**Table 2:** Group analysis results of activation in response to manual acupuncture at ST 36.

Regions	Voxels	Side	Peak MNI coordinate (mm)			*T* value (peak intensity)
			*X*	*Y*	*Z*	
Insula	308	R	45	11	11	8.63
Middle frontal gyrus	165	L	−36	50	−1	4.91
Postcentral gyrus	132	L	−60	−25	41	5.25
Lentiform nucleus	115	L	−33	−4	−1	5.92
Cerebellum	111	R	45	−73	−40	5.12
Precentral gyrus	83	L	−54	5	14	5.29
Anterior cingulate cortex	70	R	6	−7	38	4.94
Middle frontal gyrus	69	R	42	44	17	4.18
Postcentral gyrus	69	R	63	−16	23	7.22

## Data Availability

The original data used to support the findings of this study are available from the corresponding author (Bensheng Qiu, bqiu@ustc.edu.cn) upon request.

## References

[1] Li P., Qiu T., Qin C. (2015). Efficacy of acupuncture for Bell’s palsy: a systematic review and meta-analysis of randomized controlled trials. *PLoS One*.

[2] Yuan Q. L., Wang P., Liu L. (2016). Acupuncture for musculoskeletal pain: a meta-analysis and meta-regression of sham-controlled randomized clinical trials. *Scientific Reports*.

[3] Park H.-J., Chae Y., Jang J., Shim I., Lee H., Lim S. (2005). The effect of acupuncture on anxiety and neuropeptide Y expression in the basolateral amygdala of maternally separated rats. *Neuroscience Letters*.

[4] Ding S. S., Hong S. H., Wang C., Guo Y., Wang Z. K., Xu Y. (2014). Acupuncture modulates the neuro-endocrine-immune network. *QJM: An International Journal of Medicine*.

[5] Tsuruoka N., Watanabe M., Seki T., Matsunaga T., Hagaa Y. Acupoint stimulation device using focused ultrasound.

[6] Quah-Smith I., Williams M. A., Lundeberg T., Suo C., Sachdev P. (2013). Differential brain effects of laser and needle acupuncture at LR8 using functional MRI. *Acupuncture in Medicine*.

[7] Wan Y., Wilson S. G., Han J., Mogil J. S. (2001). The effect of genotype on sensitivity to electroacupuncture analgesia. *Pain*.

[8] Cevik C., Iseri S. O. (2013). The effect of acupuncture on high blood pressure of patients using antihypertensive drugs. *Acupuncture & Electro-Therapeutics Research*.

[9] Yang Y. H., Zhang D., Sa Z. Y., Huang M., Ding G. H. (2012). Development of studies on bioeffects of ultrasound-acupuncture therapy and its underlying mechanism. *Acupunct Research*.

[10] Bai L., Qin W., Tian J. (2009). Time-varied characteristics of acupuncture effects in fMRI studies. *Human Brain Mapping*.

[11] Hui K. K. S., Liu J., Makris N. (2000). Acupuncture modulates the limbic system and subcortical gray structures of the human brain: evidence from fMRI studies in normal subjects. *Human Brain Mapping*.

[12] Napadow V., Lee J., Kim J. (2013). Brain correlates of phasic autonomic response to acupuncture stimulation: an event-related fMRI study. *Human Brain Mapping*.

[13] Hui K. K. S., Liu J., Marina O. (2005). The integrated response of the human cerebro-cerebellar and limbic systems to acupuncture stimulation at ST 36 as evidenced by fMRI. *Neuroimage*.

[14] Nierhaus T., Pach D., Huang W. (2015). Differential cerebral response to somatosensory stimulation of an acupuncture point vs. two non-acupuncture points measured with EEG and fMRI. *Frontiers in Human Neuroscience*.

[15] Wu M., Sheen J. M., Chuang K. H. (2002). Neuronal specificity of acupuncture response: a fMRI study with electroacupuncture. *Neuroimage*.

[16] Quah-Smith I., Sachdev S. P., Wen W., Chen X., Williams A. M. (2010). The brain effects of laser acupuncture in healthy individuals: an fMRI investigation. *PLoS One*.

[17] Park S.-U., Shin A.-S., Jahng G.-H., Moon S.-K., Park J.-M. (2009). Effects of scalp acupuncture versus upper and lower limb acupuncture on signal activation of blood oxygen level dependent (BOLD) fMRI of the brain and somatosensory cortex. *The Journal of Alternative and Complementary Medicine*.

[18] Napadow V., Makris N., Liu J., Kettner N. W., Kwong K. K., Hui K. K. S. (2005). Effects of electroacupuncture versus manual acupuncture on the human brain as measured by fMRI. *Human Brain Mapping*.

[19] Bai Y. H. (1990). Comparison of the pain relief from four acupuncture modalities. *Shanghai Acupuncture Magazine*.

[20] Tsuruoka N., Watanabe M., Takayama S., Seki T., Matsunaga T., Haga Y. (2013). Brief effect of acupoint stimulation using focused ultrasound. *The Journal of Alternative and Complementary Medicine*.

[21] Wang S.-H., Chen Y.-T., Weng C.-S., Tsui P.-H., Huang J.-L., Chiang K.-C. (2003). A clinical therapeutic assessment for the administration of different modes of ultrasounds to stimulate the zusanli acupuncture point of hypertension patients. *Journal of Medical and Biological Engineering*.

[22] Ou H., Fang L., Bai J., Diao Q., Zhai B. (2012). Effect of ultrasound stimulation at the acupoint guanyuan on follicular development in menopausal rats. *Nan Fang Yi Ke Da Xue Xue Bao*.

[23] Thakur A., Mandal S. C., Banerjee S. (2016). Compounds of natural origin and acupuncture for the treatment of diseases caused by estrogen deficiency. *Journal of Acupuncture and Meridian Studies*.

[24] Critchley H. D., Wiens S., Rotshtein P., Öhman A., Dolan R. J. (2004). Neural systems supporting interoceptive awareness. *Nature Neuroscience*.

[25] Sojka P., Losak J., Lamos M. (2019). Processing of emotions in functional movement disorder: an exploratory fMRI study. *Frontiers in Neurology*.

[26] Petrides M., Pandya D. N. (2012). The frontal cortex. *The Human Nervous System*.

[27] Bantick S. J., Wise R. G., Ploghaus A., Clare S., Smith S. M., Tracey I. (2002). Imaging how attention modulates pain in humans using functional MRI. *Brain*.

[28] Davis K. D., Taylor S. J., Crawley A. P., Wood M. L., Mikulis D. J. (1997). Functional MRI of pain- and attention-related activations in the human cingulate cortex. *Journal of Neurophysiology*.

[29] Fang J., Jin Z., Wang Y. (2009). The salient characteristics of the central effects of acupuncture needling: limbic-paralimbic-neocortical network modulation. *Human Brain Mapping*.

[30] Dyson M., Luke D. A. (1986). Induction of mast cell degranulation in skin by ultrasound. *IEEE Transactions on Ultrasonics, Ferroelectrics and Frequency Control*.

[31] Cui X., Liu K., Xu D. (2018). Mast cell deficiency attenuates acupuncture analgesia for mechanical pain using c-kit gene mutant rats. *Journal of Pain Research*.

[32] Yoo S.-S., Lee W., Kim H. (2014). Pulsed application of focused ultrasound to the LI4 elicits deqi sensations: pilot study. *Complementary Therapies in Medicine*.

[33] Stefanello D., Valenti P., Faverzani S. (2009). Ultrasound-guided cytology of spleen and liver: a prognostic tool in canine cutaneous mast cell tumor. *Journal of Veterinary Internal Medicine*.

[34] Yao W., Yang H., Yin N., Ding G. (2014). Mast cell-nerve cell interaction at acupoint: modeling mechanotransduction pathway induced by acupuncture. *International Journal of Biological Sciences*.

[35] Yoon S.-Y., Roh D.-H., Kwon Y.-B. (2009). Acupoint stimulation with diluted bee venom (apipuncture) potentiates the analgesic effect of intrathecal clonidine in the rodent formalin test and in a neuropathic pain model. *The Journal of Pain*.

[36] Napadow V., Liu J., Li M. (2007). Somatosensory cortical plasticity in carpal tunnel syndrome treated by acupuncture. *Human Brain Mapping*.

[37] Ebenbichler G. R., Resch K. L., Nicolakis P. (1998). Ultrasound treatment for treating the carpal tunnel syndrome: randomised “sham” controlled trial. *British Medical Journal*.

[38] Goyatá S. L. T., Avelino C. C. V., Santos S. V. M. D., Souza Junior D. I. D., Gurgel M. D. S. L., Terra F. D. S. (2016). Efeitos da acupuntura no tratamento da ansiedade: revisao integrativa. *Revista Brasileira de Enfermagem*.

[39] Schunck T., Erb G., Mathis A. (2008). Test-retest reliability of a functional MRI anticipatory anxiety paradigm in healthy volunteers. *Journal of Magnetic Resonance Imaging*.

[40] Lin W.-C., Chou K.-H., Chen H.-L. (2012). Structural deficits in the emotion circuit and cerebellum are associated with depression, anxiety and cognitive dysfunction in methadone maintenance patients: a voxel-based morphometric study. *Psychiatry Research: Neuroimaging*.

[41] Maddock R. J., Buonocore M. H. (1997). Activation of left posterior cingulate gyrus by the auditory presentation ofthreat-related words: an fMRI study. *Psychiatry Research: Neuroimaging*.

[42] Huang W., Pach D., Napadow V. (2012). Characterizing acupuncture stimuli using brain imaging with FMRI-a systematic review and meta-analysis of the literature. *PLoS One*.

